# Novel Methylselenoesters Induce Programed Cell Death via Entosis in Pancreatic Cancer Cells

**DOI:** 10.3390/ijms19102849

**Published:** 2018-09-20

**Authors:** Prajakta Khalkar, Nuria Díaz-Argelich, Juan Antonio Palop, Carmen Sanmartín, Aristi P. Fernandes

**Affiliations:** 1 Division of Biochemistry, Department of Medical Biochemistry and Biophysics (MBB), Karolinska Institutet, SE-171 77 Stockholm, Sweden; ndiaz@alumni.unav.es (N.D.-A.); Aristi.Fernandes@ki.se (A.P.F.); 2 Department of Organic and Pharmaceutical Chemistry, Faculty of Pharmacy and Nutrition, University of Navarra, Irunlarrea 1, E-31008 Pamplona, Spain; japcubillo@gmail.com (J.A.P.); sanmartin@unav.es (C.S.); 3 IdiSNA, Navarra Institute for Health Research, Irunlarrea 3, E-31008 Pamplona, Spain

**Keywords:** selenium, methylselenoesters, entosis, anticancer agent

## Abstract

Redox active selenium (Se) compounds have gained substantial attention in the last decade as potential cancer therapeutic agents. Several Se compounds have shown high selectivity and sensitivity against malignant cells. The cytotoxic effects are exerted by their biologically active metabolites, with methylselenol (CH_3_SeH) being one of the key executors. In search of novel CH_3_SeH precursors, we previously synthesized a series of methylselenoesters that were active (GI_50_ < 10 µM at 72 h) against a panel of cancer cell lines. Herein, we refined the mechanism of action of the two lead compounds with the additional synthesis of new analogs (ethyl, pentyl, and benzyl derivatives). A novel mechanism for the programmed cell death mechanism for Se-compounds was identified. Both methylseleninic acid and the novel CH_3_SeH precursors induced entosis by cell detachment through downregulation of cell division control protein 42 homolog (CDC42) and its downstream effector β1-integrin (CD29). To our knowledge, this is the first time that Se compounds have been reported to induce this type of cell death and is of importance in the characterization of the anticancerogenic properties of these compounds.

## 1. Introduction

Pancreatic ductal adenocarcinoma is an extremely aggressive neoplasm and one of the cancers with the poorest prognosis, with a five-year survival of only 8% [1]. In addition, it is predicted to become the second leading cause of cancer-related death by 2030 [2]. Late diagnosis in advanced cancer stages due to a lack of prior symptomatology and the poor efficiency of actual therapeutics are the main causes. Drug resistance in pancreatic cancer is largely caused by an active stroma contributing to tumor progression [3]. Therefore, developing new therapeutic strategies has become an urgent need.

Modulation of redox homeostasis in cancer cells has emerged as a new opportunity for tumor intervention. Induction of reactive oxygen species (ROS) by these compounds may affect all the redox dependent pathways in the cell, which can be detrimental to cells. Antioxidant enzymes are often induced to eliminate elevated ROS production. Due to metabolic transformation, cancer cells have an increased and maximized antioxidant capacity in order to evade the ROS-induced cell death. For instance, the expression of mutant oncogenic KrasG12D is commonly present in pancreatic ductal adenocarcinoma (PDAC), resulting in an elevated basal state of the transcription factor, nuclear factor E2-related factor 2 (NRF2) to mount an antioxidant response [4,5]. Therefore, even a slight additional ROS induction, using redox modulators, would lead to the killing of cancer cells [6,7], and provides an interesting therapeutic approach that has been established as a means of successful anti-cancer therapy [8,9,10,11].

Redox modulating selenium (Se) compounds have gained substantial attention in the last decade as potential cancer therapeutic agents [12]. Several Se compounds have shown high selectivity and sensitivity in malignant cells [13]. Depending on the compound use, they have been reported to induce different types of cell death, including apoptosis, autophagy, necrosis, or necroptosis. 

Importantly, along with their active metabolites that execute their biological activity, the dosage and chemical form of Se compounds highly determine their efficacy [12]. Methylselenol (CH_3_SeH) is considered a key metabolite in the anticancer activity of Se compounds. However, the in situ production or alternatively, the use of precursors, is required due to the high reactivity and volatility of this molecule.

**Figure 1:**
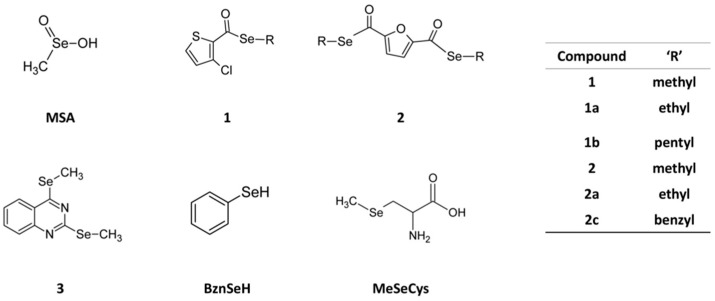
Chemical structures of the compounds. MSA, and compounds **1** and **2** were the primary focus of this study, whereas remaining compounds were used for comparative analysis in some experiments. MSA: methylseleninic acid; R: substituent; BznSeH: benzeneselenol; MeSeCys: methylselenocysteine.

In search of novel CH_3_SeH precursors, we previously synthesized a series of methylselenoesters that were active (GI_50_ < 10 µM at 72 h) against a panel of cancer cell lines [14]. Herein, we studied the mechanism of action of the two lead compounds with the additional synthesis of new analogs (ethyl, pentyl, and benzyl derivatives) (Figure 1). This study uncovers a novel cell death mechanism for these Se-compounds as entosis inducers. Entosis was first described under anchorage-independent conditions and the loss of β1-integrin (CD29) signaling [15]. However, it has also been described in adherent cells [16,17,18] and recently, aberrant mitosis [16] and glucose deprivation [19] have been identified as other possible triggers. 

During entosis, the stiffer cell (hereafter target cell) actively participates in its own internalization, via adherent junctions and the actin cytoskeleton that play a pivotal role in this process. Ultimately, the target cell is killed through lysosomal enzyme-mediated degradation, using the autophagy machinery, but independent of autophagosome formation [20]. The death subroutine might swift to apoptosis in the absence of autophagy-dependent nutrient recycling, or eventually, the internalized cell might divide or be released [21].

Methylseleninic acid (MSA) and the novel CH_3_SeH precursors induce cell detachment through downregulation of cell division control protein 42 homolog (CDC42) and its downstream effector CD29 [22]. Cell-cell adhesion molecules such as N-cadherin were upregulated after treatment and facilitated cell clustering, which finally ended with cell-in-cell invasion and the degradation of the inner cell. To our knowledge, this is the first time that Se compounds have been reported to induce this type of cell death.

## 2. Results

### 2.1. MSA, and Compounds ***1*** and ***2*** Reduce Panc-1 Cell Viability Both in 2D and 3D Cultures

**Figure 2:**
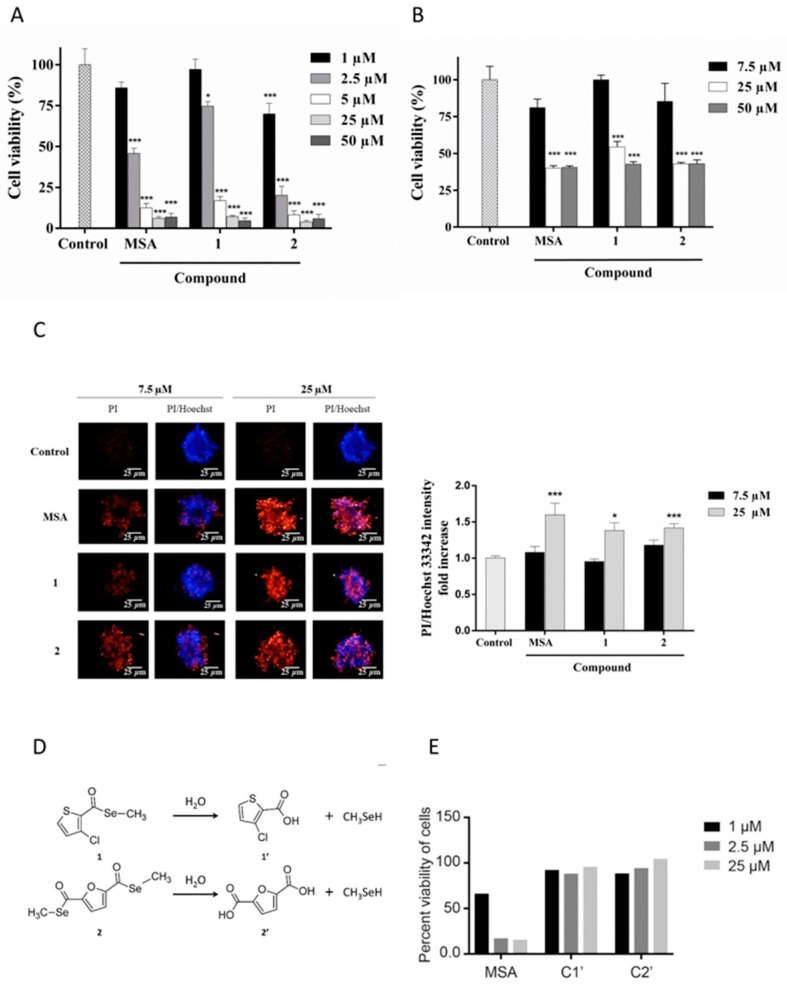
Compounds **1** and **2** and MSA decrease cell viability in 2D and 3D Panc-1 cultures. (**A**) Panc-1 cells (2D cultures) were treated with different concentrations of the compounds for 72 h followed by the determination of cell viability by the MTT (3-(4,5-Dimethylthiazol-2-yl)-2,5-Diphenyltetrazolium Bromide) assay. Results represent mean ± SEM of at least three independent experiments performed in quadruplicate. (**B**) Panc-1 spheroids (3D cultures) were treated with different concentrations of the compounds for 72 h, after which cell viability was determined using the acid phosphatase (APH) assay. Results represent mean ± SEM of at least three independent experiments performed in quadruplicate. (**C**) Representative confocal images of Panc-1 spheroids stained with Hoechst 33342 and PI after 72 h treatment with 7.5 µM and 25 µM of respective compounds. 10× objective magnification images were acquired from the Operetta^®^ High-Content Imaging System and processed by Colombus™ analysis software. The adjacent graph represents a quantitative analysis of PI/Hoechst fluorescence. Results represent mean ± SEM (*n* = 4). (**D**) Potential hydrolysis reaction of compounds **1** and **2**. (**E**) 2D cell viability after treatment with the corresponding carboxylic acid for 72 h. Statistical significance compared to control: * *p* < 0.05, *** *p* < 0.001.

Initial characterization of the compounds was performed through viability assays in 2D and 3D cultures of Panc-1 cells, given that 3D cultures have been demonstrated to mimic tumor behavior more efficiently than traditional monolayer (2D) cultures. Panc-1 cells were treated with increasing concentrations of MSA, and compounds **1** or **2** for 72 h. Cell viability was then determined. All three compounds were cytotoxic, with compound 2 being the most potent compound in 2D cultures. The compounds had IC50 values in the low micromolar range in 2D cultures (2.28, 3.31, and 1.43 µM for MSA, and compounds **1** and **2**, respectively). However, cells grown as spheroids (3D) were consistent with previously reported data [23], and more resistant and higher doses of the compounds were required to reduce cell proliferation and induce cell death (Figure 2A,B).

To further study the induced cell death in 3D cultures, spheroids were stained with Hoechst and propidium iodide (PI) after 72 h treatment. While Hoechst stains the nucleus of all cells, PI only penetrates and stains damaged membranes of dying cells. As shown in Figure 2C, the three compounds were not only able to induce cell death, but the cell death was observed in the core of the spheroid, suggesting that these compounds were able to reach to the core of the sphere.

The selenoester entity could be easily hydrolyzed by a nucleophile such as water, rendering the corresponding carboxylic acids and releasing CH_3_SeH, which is believed to be a key molecule in Se activity (Figure 2D). To exclude the possibility that the toxicity was from the linked moieties, the analog carboxylic acids of compounds **1** (**1’**) and **2** (**2’**) were selectively tested as a proof-of-concept. As seen in Figure 2E, they did not induce any cell death compared to the Se-containing molecules.

### 2.2. MSA, and Compounds ***1*** and ***2*** Induce Cell Detachment and Compromise Reattachment Abilities by Promoting an Aberrant Adhesive Repertoire

**Figure 3:**
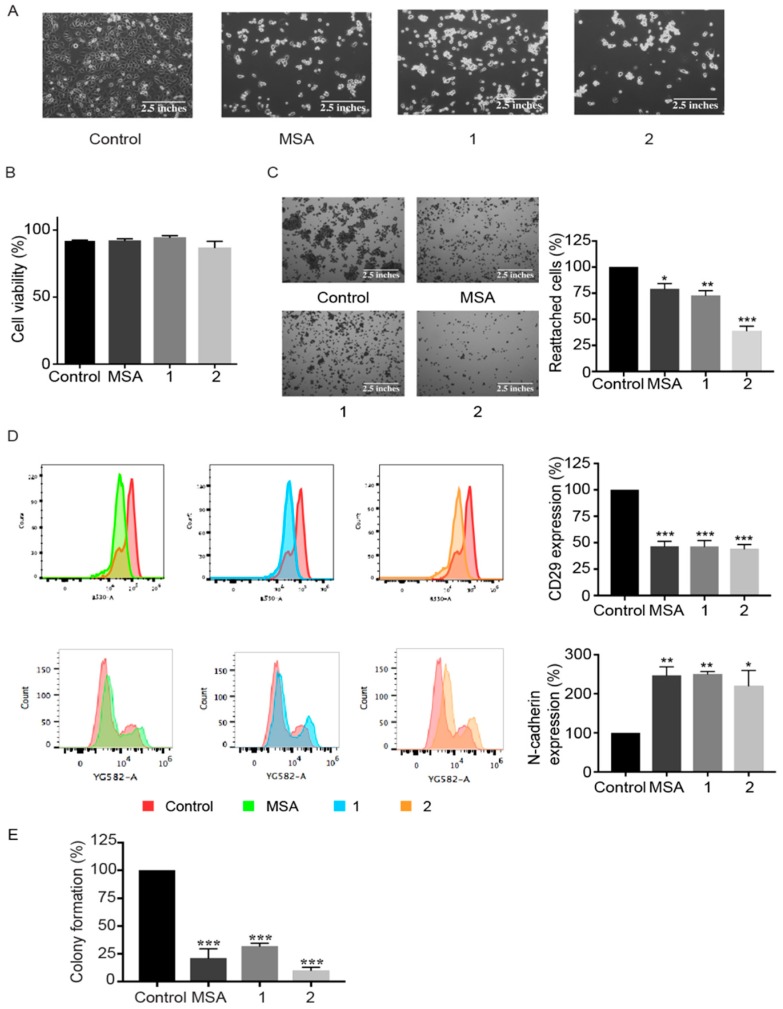
Compounds **1** and **2** and MSA induce loss of cellular adhesion prior to cell death and impair the colony forming ability. Panc-1 cells were treated with 5 µM of MSA, or compounds **1** or **2** for 24 h. (**A**) Representative phase-contrast images of treatment-induced cell detachment with respective compounds. (**B**) The viability of the floating cells was assessed using a trypan blue exclusion assay. (**C**) Adhesion assay. After 24 h of treatment with respective compounds, the non-adherent but viable cells, were collected and an adhesion assay was performed for 3 h, following which the adherent cells were stained with Coomassie Brilliant Blue. Representative phase-contrast microscopic images and graphical representation of percentage of non-adherent cells reattaching the tissue culture treated plates. Error bars indicate mean ± SEM of three biological replicates. (**D**) The expression levels of adhesion proteins, CD29, and N-Cadherin, post 24 h treatment with respective compounds as analyzed by flow cytometry and its graphical representation. Error bars indicate mean ± SEM of three biological replicates. (**E**) Clonogenic assay. Post 24 h treatment, with respective compounds, the non-adherent cells were collected and re-seeded in 24 well plates to check for the colony forming ability of these cells. Reduced colony forming ability indicates cell death. Error bars indicate mean ± SEM of three biological replicates. Statistical significance as compared to control * *p* < 0.05, ** *p* < 0.01, *** *p* < 0.001.

In order to study the early effects of this particular cell death, a concentration of 5 µM of respective compounds was chosen for further experiments in 2D cultures. Post 6 h treatment of Panc-1 cells, morphological changes like rounding of the cells and cellular detachment from culture flasks were observed. At 24 h, almost all the cells were detached, had acquired a refringent morphology, and were grouped in a grape-like manner (Figure 3A). Trypan blue exclusion, however, indicated that the floating cells were still alive at that particular time point (Figure 3B). To examine if the aberrant cellular detachment was irreversible, an adhesion assay was performed wherein the floating cells were washed to remove traces of the compounds and reseeded in fresh medium. The cells were then allowed to reattach to culture flasks for 3 h. Nevertheless, their reattachment abilities after treatment were observed to be compromised, with a clear loss of ability to re-adhere, especially in the case of compound **2** (Figure 3C).

As a next step, the effect of respective compounds on different cellular adhesion markers was analyzed. Post 24 h treatment, the expression of CD29, known to mediate adhesion to the extracellular matrix [24], was significantly reduced, as observed after flow cytometry analysis (Figure 3D), explaining the loss of cellular adhesion caused by these compounds. Moreover, the expression of N-cadherin, a cell-cell adhesion marker [25], showed a considerable increase after treatment with respective compounds, which explains the observed grape-like cellular clumping after detachment (Figure 3D).

In order to assess the fate of the detached cells, i.e., if they were able to recover or eventually go into the cell death mode, a clonogenic assay was performed. As illustrated in Figure 3E, post 24 h treatment with respective compounds, the cells displayed a significant decrease in colony formation compared to the control.

### 2.3. MSA, Compounds ***1*** and ***2*** Induce Entosis.

Two well described cell death pathways that have been reported to be initiated by the loss of cell adhesion are anoikis and entosis. Whereas anoikis is triggered exclusively upon adhesion loss and is coursed through caspase activation, entosis is characterized by active cell invasion, leading to endophagocytosis and the formation of cell-in-cell structures, and has been described both in suspension and adherent cells. 

To distinguish the programmed cell death mode, we analyzed the expression of total poly (ADP-ribose) polymerase 1 (PARP) and cleaved PARP, wherein MSA and compounds **1** and **2** slightly increased the 89 kD cleaved fragment at 72 h (Supplementary Figure S1A). PARP has been reported to be cleaved by caspases, cathepsins, and calpains [26,27,28,29]. In order to rule out the possibility of apoptosis, the expression of caspase 9 and cleaved caspase 9 (upstream marker for apoptosis) was analyzed after 48 h treatment with these compounds. We observed no expression of cleaved caspase 9, suggesting that no activation of the caspase cascade was induced (Supplementary Figure S1A). Additionally, cells were treated with the broad pancaspase inhibitor z-VAD-fmk, along with respective compounds. Treatment with z-VAD-fmk did not prevent the cellular detachment, as well as cell death, induced by these compounds, as observed by brightfield microscopy and the trypan blue exclusion assay, respectively (Supplementary Figure S1B,C), suggesting a caspase-independent mechanism.

Furthermore, the expression of cathepsins, a structurally and catalytically distinguished class of proteases, was checked. A context-depending role has been described for cathepsins, with either tumor-promoting or suppressing activities. They have not only been reported to function as apoptotic mediators, but also to be related to entosis [15] and cell cannibalism [30].

**Figure 4:**
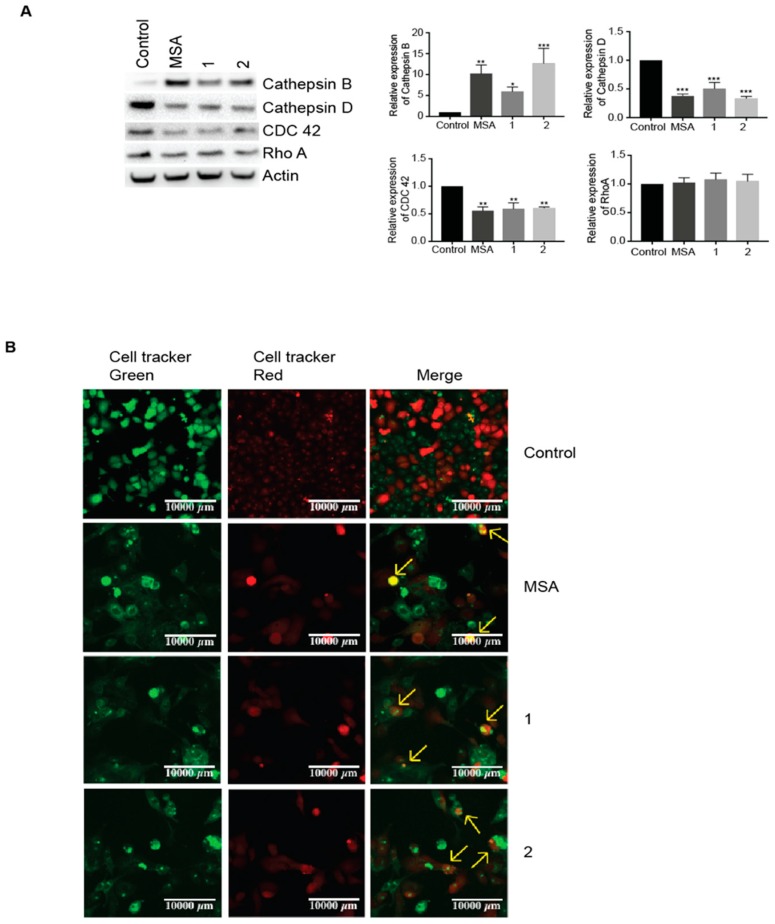
MSA, and compounds **1** and compound **2** induce entosis in Panc-1 cells. (**A**) Western blot analyses of Cathepsin B, Cathepsin D, CDC42, and Rho A upon treatment with MSA or compound **1** or compound **2** for 24 h. Beta actin was used as a loading control. The corresponding graphs display a quantitative analysis of western blots performed using the ImageJ program and GraphPad Prism software. Error bars indicate mean ± SEM of three biological replicates. (**B**) Panc-1 cells stained with CellTracker Red or Green and further mixed (1:1) were treated with MSA or compound **1** or compound **2** for 18 h, followed by live imaging using a confocal microscope, Zeiss LSM800. The arrows indicate cell-in-cell formations i.e., cells undergoing entosis. Statistical significance as compared to control * *p* < 0.05, ** *p* < 0.01, *** *p* < 0.001.

Both cathepsin B (CatB) and cathepsin D (CatD) have been reported to play an important role in entosis [15,20,31]. Increased expression of CatB was observed, indicating that lysosomal degradation is implied in cell death induced by these compounds. Unexpectedly, CatD levels were downregulated (Figure 4A). Another family with a prominent role in entosis is the Rho family of GTPases, master regulators of the actin cytoskeleton. Therefore, the protein levels of CDC42 and RhoA were determined. Whereas CDC42 levels were decreased, RhoA levels remained unchanged (Figure 4A). To further confirm entosis, cell fate was tracked once detached. Cells were labeled with green or red fluorescent dyes, seeded, and treated with the compounds. Visualization by confocal microscopy revealed cell-in-cell internalization (Figure 4B). A time-lapse experiment also recorded live confirmed morphological changes during the formation of cell-in-cell structures and the ultimate degradation of the target cell (Supplementary videos 1–3).

### 2.4. Cell Detachment Is Not Restricted to Selenomethylated Compounds and Does Not Correlate with the Cytotoxic Potential of the Compound

In order to distinguish the type of Se compound that could cause this phenomenon, other commercial Se derivatives together with other newly synthesized analogs of compounds **1** and **2** were analyzed. To evaluate if this effect was exclusive to methylated forms of Se or unrestricted to other alkyl or aromatic derivatives, the ethyl derivative for compounds **1** and **2** (**1a** and **2a**, respectively), the pentyl derivative for compound **1** (**1b**), and the benzyl analog for compound **2** (**2c**), were synthesized to cover different alkyl lengths and additional substituents. Methylselenocysteine (MeSeCys) was also selected as another CH_3_SeH precursor and benzeneselenol (BznSeH) as an additional aromatic selenol for a comparative analysis. In addition, compound **3** was chosen, a previously synthesized selenide in our laboratory, as a proof-of-concept compound without a labile bond between the core of the molecule and the methylseleno residue [32], and therefore less prone to release it (Figure 1). 

**Figure 5:**
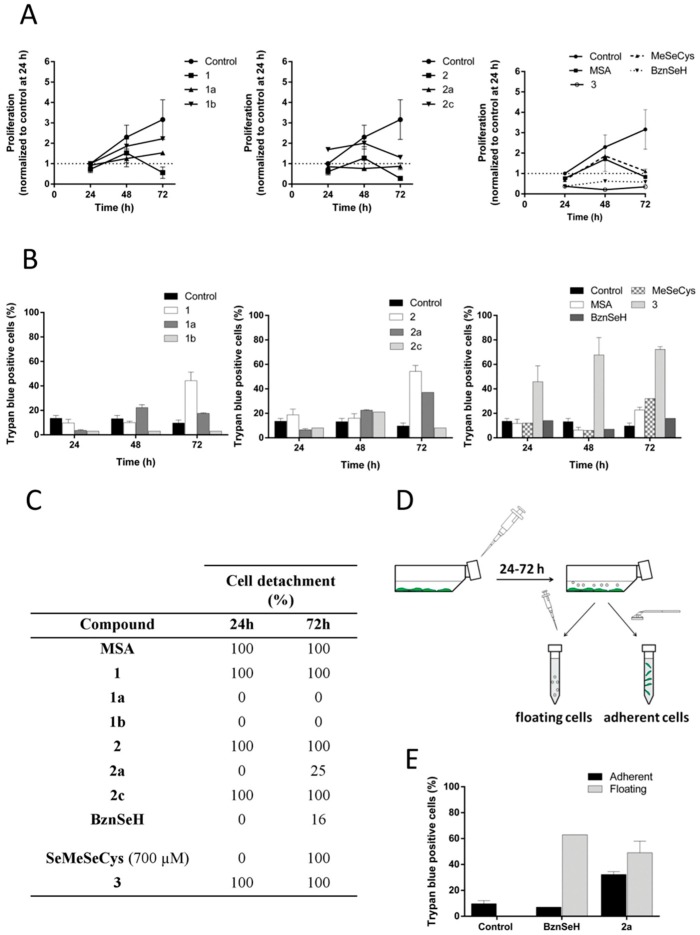
Evaluation of the antiproliferative, cytotoxic, and de-adhesive properties of other Se analogs. Cells were seeded and incubated for 24 h before starting treatments with the compounds. Cell proliferation (**A**) and cell death based on Trypan blue exclusion (**B**) were analyzed after treatment with a 5 µM dose. For MeSeCys, a 700 µM dose was used. For BznSeH and compound **2a**, inducing a floating and attached population, proliferation, and cell death were calculated without taking into account the two populations in this case. (**C**) Cell detachment quantification after treatment. (**D**) Procedure scheme to evaluate the attached and floating population. Floating cells were collected with a pipette and remaining cells were considered as attached and slightly scrapped. (**E**) Cell death comparison in the floating and adherent populations induced by compound **2a** and BznSeH.

First, cell proliferation and cell death were evaluated. As illustrated in Figure 5A, all the compounds were able to reduce cell proliferation. However, a longer chain or the substitution with a benzyl residue impaired the cytostatic activity of the compounds. In general trends, and considering the 72 h time point, the potency to reduce proliferation decreased according to the following order: methyl > ethyl > pentyl or benzyl. Compound **2a** stopped proliferation at 24 h, while the methylated analog (**2**) achieved a reduction at 72 h. BznSeH and compound **3** had the highest cytostatic potential, with both of them inhibiting proliferation at 24 h of treatment.

The methyl derivatives were more cytotoxic at 72 h than analogs with a longer alkyl chain or the benzyl moiety (Figure 5B). Almost all the compounds showed similar activity at 24 and 48 h, with the exception of compound **1b**, which was not cytotoxic. BznSeH, which was highly cytostatic, did not induce noteworthy cell death, with only 15% of dead cells at 72 h. MeSeCys, which has been reported to have a similar activity to MSA in vivo, required a considerably higher concentration to achieve similar cell death induction in vitro, due to the need of metabolic processing to release CH_3_SeH, consistent with previous reports [33]. Compound **3**, on the other hand, was the most potent compound, inducing cell death at 24 h treatment. 

In addition to cell proliferation and cell death, the ability of the compounds to induce cell detachment, and ultimately the same cell death mechanism as the methyl analogs, was analyzed. Most of the compounds completely detached the cells or completely remained ineffective, as illustrated in Figure 5C. However, some compounds induced two populations, and in this case, the procedure schematized in Figure 5D was followed. Compounds **1a** and **1b** were unable to detach cells. whereas compounds **2c** and **3** had detached all the cells at 24 h. MeSeCys detached all the cells at 72 h, and a concomitant increase in cell death was observed at that time point. However, cell detachment potential was not correlated with cell death induction in the case of compound **2c**, which was almost innocuous. BznSeH and compound **2a** induced mixed populations, with attached and floating cell fractions. (Figure 5D,E). Nevertheless, they had considerably less detachment potential than the methylated analogs, with only 16 and 24% of detached cells at 72 h, respectively.

## 3. Discussion

In this study, we demonstrate that MSA and two novel methylselenoesters induce entosis after provoking cell detachment in Panc-1 cells, revealing a new and unexplored cell death mechanism for Se compounds. 

Compounds **1** and **2** and MSA reduced cell proliferation in both 2D and 3D cultures. Treatment with the compounds led to a unique phenotype, characterized by changes in morphology and cell detachment from the culture plate prior to cell death. Detached cells were alive at 24 h, but their reattachment capability and the colony forming ability had been dramatically compromised. We dismissed the possibility that the compounds were promoting anchorage-independent survival, and instead induced cellular death, as confirmed by the MTT assay and the expression of cleaved PARP in 2D cultures and PI staining in 3D spheroids.

Cell adhesion is gaining more attention due to its implication in cancer metastasis and progression, in addition to drug resistance. Importantly, these results are in accordance with recent investigations revealing that MSA targeted adhesion molecules in a leukemic cancer cell line, whereas inorganic selenite affected other gene sets, indicating an interesting type-dependent effect of Se compounds [34]. To further confirm the compound-induced adhesion disturbance, levels of different adhesion molecules were screened 24 h after treatment. We found that the expression of CD29 was significantly reduced. This integrin has been linked to gemcitabine resistance and a poor outcome in pancreatic cancer [35]. Moreover, its knockdown has been reported to inhibit cell adhesion, migration, proliferation, and metastasis of pancreatic cancer, unveiling CD29 as a potential therapeutic target [36]. 

The loss of CD29 signaling and consequent detachment from culture plate trigger entosis [16]. Entosis is primarily the engulfment of one live cell into another live cell. In our study, the detached cells post 24 h treatment were observed to be viable. Also, the formation of adherent junctions has been shown to be crucial for entosis initiation [15]. This kind of cell-cell contacts are mediated though cadherins, which are calcium-dependent molecules that play central roles in cancer progression. We found increased N-cadherin levels, which could explain cell clumping after detachment and the ultimate invasion of one cell into another. Although E-cadherin usually forms adherens junctions in epithelial cells, the coexpression of E- and N-cadherin has been reported in adherens junctions of endoderm-derived epithelial tissues and tumors, such as pancreatic ducts [37]. In addition, Panc-1 cells express very low basal levels of E-cadherin and, according to Cano et al. [38], it cannot be discarded that pancreatic homotypic cell-in-cell formation might rely on N-cadherin-mediated cell contacts. Although N-cadherin is usually linked to a more aggressive phenotype, it has been reported as a tumor suppressor in some types of cancers [39,40].

In addition, an upstream regulator of CD29 [22] and member of the Rho family of GTPases, CDC42, was also observed to be downregulated after treatment. CDC42 is overexpressed by 21% in pancreatic cancer [41] and the depletion of CDC42 enhances mitotic deadhesion and depends on Rho A activation in human bronchial epithelial cells [16]. Although it plays a crucial role in adherent entosis, it was reported to have no effect on suspension cells [16]. Consequently, treatment with the Se compounds affects CDC42 expression and mediates cell detachment through CD29 regulation.

Entotic cells mainly die through lysosomal-dependent pathways, although a swift to apoptosis can occur. In a floating population, different types of cell death have been reported to coexist [42].

Herein, we found that cell death induced by these Se compounds was caspase-independent, with a slight increase in PARP cleavage. We found increased levels of CatB in cell-in-cell structures undergoing entotic death, in concordance with previous reports [15]. By contrast, CatD, an interplayer between autophagy and apoptosis, was clearly downregulated. CatD can function as an anti-apoptotic mediator by increasing autophagy, revealing its two-faceted role [43]. In addition, CatD enhances anchorage-independent cell proliferation [44], and it is therefore quite interesting that it becomes down-regulated by compounds inducing cell detachment. Although cathepsins can mediate apoptosis, high levels of cathepsins have also been related to cancer progression. Pancreatic cancer patients, for instance, display a higher CatD concentration than healthy controls [45], and besides, elevated levels have been shown to promote cell dissemination in pancreatic cancer in vivo [46].

Cell detachment could be caused by CH_3_SeH, one of the key metabolites in Se cytotoxicity, which has been reported to cause cell detachment in different cancer cell lines [47,48], along with a decrease in CD29 expression [48]. MSA is a penultimate precursor and compounds **1** and **2** bear this moiety. However, we ruled out that this effect was exclusive to the methylated form of Se, given that other compounds were able to induce the same phenotype. Lengthening the alkyl chain or the substitution over an arylselenol in general dramatically decreased the percentage of detached cells. However, the substitution of methyl for benzyl (compound **2c**) induced similar deadhesive effects. Intriguingly, despite induced cell detachment by this compound, it did not lead to cell death. The decreased cytotoxic effects are consistent with previous reports, showing the impaired cytotoxic activity of selenobenzyl derivatives with respect to their corresponding methylated analogs [49]. Hence, it is clear that detachment per se does not trigger death signaling, and it will be interesting to investigate the additional signaling pathways that the methyl and benzylseleno moieties are differentially able to activate, in order to avoid anchorange-independent cell growth.

In summary, we report a novel mechanism of action for MSA and two methylselenoesters: the induction of cell detachment through CDC42 and CD29 down-regulation leading to cell-in-cell formation (entosis) and death of the inner cell. However, these compounds need to be further evaluated in in vivo studies to gain an in-depth insight into their administration, hepatic metabolism for bioavailability and absorption, distribution, metabolism, and excretion properties. Additionally, the therapeutic potential of these compounds would be governed by the balance between their toxicity and efficacy profiles. Therefore, further research to fully dissect the relationship between structure, detachment abilities, and cell death induction of organic Se derivatives is required in order to understand the complex Se biochemistry.

## 4. Materials and Methods 

### 4.1. Cell Culture

Panc-1 cells were obtained from the American Type Culture Collection (ATCC) and cultured in DMEM:F12 (Gibco™, ThermoFisher Scientific, Paisley, Scotland), 10% FBS (HyClone™, GE Healthcare Life Sciences, Logan, UT, USA), and 1% glutamine (Gibco) at 37 °C under 5% CO_2_. The 3D spheroids were cultured following the protocol described by Longati et al. [23]. Briefly, phenol red-free DMEM:F12 (Gibco™, ThermoFisher Scientific, Paisley, Scotland), 10% FBS (HyClone™, GE Healthcare Life Sciences, Logan, UT, USA), and 0.24% methylcellulose were used. On day 0, 2500 Panc-1 cells in 50 µL volume were seeded in a low adherent 96-well round bottom microplate (Falcon™, ThermoFisher Scientific, Stockholm, Sweden). On day 4, treatments were added, diluted in 50 µL of medium.

### 4.2. 2D Viability Assay

Cell viability after treatment was assessed by the MTT (3-(4,5-dimethylthiazol-2-yl)-2,5-diphenyltetrazolium bromide) (Sigma Aldrich^®^, Stockholm, Sweden) assay. 6000 Panc-1 cells were seeded in 96-well plates. Cells were treated with increasing concentrations of the compounds. Dilutions of the compounds in cell medium were freshly prepared from a 0.01 M stock in DMSO. After 72 h treatment, 50 µL of MTT solution in PBS (2 mg/mL) was added and cells were incubated at 37 °C under 5% CO_2_ for 4 h. Medium was removed and 150 µL of DMSO was added to dissolve the formazan crystals. Absorbance was read at 590 nm in a VersaMax microplate reader (Molecular Devices, San Jose, CA, USA). Viability is expressed as the percentage of untreated cells.

### 4.3. 3D Viability Assay

3D viability after 72 h treatment was analyzed with the acid phosphatase assay, following a previously described protocol [23]. Briefly, 70 µL of medium was carefully removed and 60 µL of PBS along with 100 µL APH buffer (1.5 M sodium acetate pH = 5.2, 0.1% TritonX-100) containing a final concentration of freshly prepared 2 mg/mL p-nitrophenyl phosphate were added. Cells were incubated for 5 h at 37 °C under 5% CO_2_ and then 10 µL of NaOH 1M was added to stop the reaction. Absorbance was read at 405 nm in a VersaMax microplate reader (Molecular Devices, San Jose, CA, USA). Viability is expressed as the percentage of untreated cells.

### 4.4. Fluorescent Staining

Spheroid formation was developed in a Gravity Trap^TM^ ULA plate (InSphero Europe GmbH, Waldshut, Germany), following the manufacturer’s protocols. Briefly, on day 0, the plate was pre-wetted with 40 µL of medium before seeding 2000 Panc-1 cells in 75 µL phenol red-free DMEM:F12, 10% FBS, and 1% glutamine. The plate was centrifuged for 2 min at 250× *g*. On day 4, cells were treated, adding 25 µL of the corresponding compound in medium. Dilutions were freshly prepared from a 0.1 M DMSO stock. On day 7, cells were stained with 1 µM Hoechst 33342 (Molecular Probes^®^, Life Technologies™, Eugene, OR, USA) for 2 h, and 2 µM PI (Molecular Probes^®^, Life Technologies™, Eugene, OR, USA) for 1 h at 37 °C under 5% CO_2_. Spheroids were then carefully washed once with PBS and fixed with paraformaldehyde (4%) at RT. Imaging was performed on the Operetta^®^ High-content Imaging System (PerkinElmer, San Jose, CA, USA) (confocal mode, 10× objective magnification, 0.3 objective NA, 35 µm focus height) and processed by the Colombus™ (PerkinElmer, San Jose, CA, USA) analysis software.

### 4.5. Western Blotting

Protein lysate containing 20 µg of proteins was separated on a Bolt 4–12% Bis-Tris Gel (Novex^TM^, ThermoFisher Scientific, Goteborg, Sweden) and transferred to a nitrocellulose membrane using the iBlot Gel Transfer Device (ThermoFisher Scientific, Goteborg, Sweden). Incubation with primary antibody (Cathepsin B (D1C7Y), Cell Signaling, Leiden, The Netherlands, Catalog no. 31718; Cathepsin D, BD Biosciences, San Jose, CA, USA, Catalog no. 610800; CDC42, Abcam, Cambridge, UK, Catalog no. ab155940; RhoA (67B89), Cell Signaling, Leiden, The Netherlands, Catalog no. 2117; PARP, Cell Signaling, Leiden, The Netherlands, Catalog no. 9542; Caspase 9, Bioss, Nordic BioSite, Stockholm, Sweden, Catalog no. bs-0049R; beta actin, Sigma-Aldrich, Stockholm, Sweden, Catalog no. A5441) diluted in TBST containing 3.5% bovine serum albumin (BSA) was done overnight at 4 °C. Secondary antibodies, goat anti-rabbit IgG HRP (Southern Biotech, Stockholm, Sweden Catalog no. 4030-05), or goat anti mouse IgG HRP (Southern Biotech, Stockholm, Sweden Catalog no. 1030-05) were incubated for 1 h. Membranes were developed using the AmershamTM ECLTM Start Western Blotting Detection Reagent (GE Healthcare Life Sciences, Logan, UT, USA) and bands were visualized using the Bio-Rad Quantity One imaging system (Bio-Rad, Stockholm, Sweden). Images were quantified using ImageJ software.

### 4.6. Adhesion Assay

0.5 × 10^6^ cells were seeded in 25 cm^2^ flasks and incubated at 37 °C under 5% CO_2_ 24 h. Media was changed and cells were treated with 5 µM of compounds, after which floating cells were collected, centrifuged, and seeded at a density of 40,000 cells in 400 µL of fresh medium/well in a 24-well plate. The control cells were scrapped and seeded at the same density in 24-well plates. The cells were allowed to adhere to the surface of the plates. Cells were incubated at 37 °C, 5% CO_2_ for 3 h, when 95% of the control cells adhered to the plate, after which they were fixed using 4% paraformaldehyde (PFA). The cells were further stained with 200 µL Coomassie blue staining solution (0.2% Coomassie Blue Brilliant R-250, 10% Acetic Acid and 40% Methanol) for 1 h at room temperature. The cells were then washed with PBS and further incubated for 1 h with 0.5 mL elution buffer (0.1 N NaOH and 50% Methanol). Furthermore, 0.5 mL of developing solution containing 10% Trichloroacetic acid (TCA) was added into the wells. Following this, 200 µL of the mix was transferred to a 96-well plate and further absorbance was read at 595 nm using the plate reader Infinite^®^ M200 Pro, Tecan, Mannedorf, Switzerland. 

### 4.7. Flow Cytometry

0.5 × 10^6^ cells were seeded in 25 cm^2^ flasks and allowed to attach for 24 h. After that, medium was replaced and cells were treated with the corresponding compounds or vehicle (DMSO) for 24 h. Cells were collected, washed with PBS-staining buffer (1% BSA, 0.01% NaN_3_, 1% FBS), and stained for 30 min at 4 °C and darkness in 50 µL PBS-staining buffer with the corresponding antibody: CD29/integrin 1-β (FITC conjugate, clone MEM-101A, Life Technologies, Eugene, OR, USA), CD325/N-cadherin (PE conjugate, clone 8C11, Life Technologies, Eugene, OR, USA). Cells were washed once with PBS-staining buffer and resuspended in fixation buffer (PBS, 1% paraformaldehyde, 2% FBS) until being read in a BD FACSCalibur™ (BD Biosciences, San Jose, CA, USA).

### 4.8. Clonogenic Assay 

0.5 × 10^6^ cells were seeded in 25 cm^2^ flasks and incubated at 37 °C under 5% CO_2_ for 24 h. Media was changed and cells were treated with 5 µM of compounds for 24 h, after which floating cells were collected, centrifuged, and seeded at a density of 1000 cells in total volume of 2 mL/well in a six-well plate. The control cells were checked for colony formation for five days. A group of 50 cells were considered as one colony. The plates were later stained with crystal violet according to Franken et al. [50].

### 4.9. Chemical Synthesis

The NMR spectra (^1^H and ^13^C) were recorded on a Bruker 400 Ultrashield^TM^ spectrometer (Rheinstetten, Germany) and are provided in the supplementary material. The samples were solved in CDCl_3_ and TMS was used as an internal standard. IR spectra were obtained on a Thermo Nicolet FT-IR Nexus spectrophotometer (Thermo Nicolet, Madison, WI, USA) using KBr pellets for solids or NaCl plates for oil compounds. The HRMS spectra were recorded on a Thermo Scientific Q Exactive Focus mass spectrometer (Thermo Scientific™, Waltham, MA, USA) by direct infusion. For TLC assays, Alugram SIL G7UV254 sheets (Macherey-Nagel; Düren, Germany) were used. Column chromatography was performed with silica gel 60 (E. Merck KGaA, Darmstadt, Germany). Chemicals were purchased from E. Merck KGaA (Darmstadt, Germany), Panreac Química S.A. (Montcada i Reixac, Barcelona, Spain), Sigma-Aldrich Quimica, S.A. (Alcobendas, Madrid, Spain), and Acros Organics (Janssen Pharmaceuticalaan, Geel, Belgium).

#### 4.9.1. Procedure for Compounds **1** and **2**

Compounds **1** and **2** were synthesized as described in our previous work [14], under the references **5** and **15**, respectively.

##### Procedure for Compounds **1a**, **2a**, **1b** and **2c**

The chemical synthesis was carried out following an already described procedure [51,52] with some modifications. Briefly, the corresponding carboxylic acid was chlorinated by a reaction with SOCl_2_. Se powder reacted with NaBH_4_ (1:2) in water or ethanol (1:1) and N_2_ atmosphere to form NaHSe. The corresponding acyl chloride dissolved in *N*,*N*-dimethylformamide (2 mL) or chloroform (2 mL) was then added and the reaction was stirred at room temperature until the reaction took place (20 min–3.5 h). The reaction was followed by IR or TLC. The mixture was filtered and the intermediate was further alkylated with the corresponding halide until discoloration of the mixture. The product was extracted with methylene chloride and dried over Na_2_SO_4_. The solvent was eliminated under rotatory evaporation and the residue was purified through column chromatography. 

##### Ethyl 3-Chlorothiophen-2-Carboselenoate (**1a**)

From 3-chlorothiophen-2-carboxylic acid (1.5 mmol), Se powder (1.5 mmol), NaBH_4_ (3 mmol), and ethyl iodide (1.5 mmol). A yellow oil was obtained, which was further purified through column chromatography using methylene chloride as the eluent. Yield: 11%. ^1^H NMR (400 MHz, CDCl_3_): δ 1.52 (t, 3H, –CH_3_, *J*_CH3-CH2_ = 7.5 Hz), 3.11 (q, 2H, –CH_2_–), 7.06 (d, 1H, H_4_ *J*_4-5_ = 5.3 Hz), 7.54 ppm (d, 1H, H_5_). ^13^C NMR (100 MHz, CDCl_3_): δ 15.7 (–CH_3_), 20.5 (–CH_2_–), 128.0 (C_4_), 130.6 (C_2_), 131.0 (C_5_), 137.7 (C_3_), 184.3 ppm (–C=O). IR (KBr): ν 3105 (w, C−H_arom_), 2962–2867 (s, C−H_aliph_), 1649 cm^−1^ (s, −C=O). HRMS calculated for C_7_H_8_ClOSSe (M + H): 254.91441, found: 254.91418.

##### Diethyl 2,5-Furandicarboselenoate (**2a**)

From 2,5-furandicarboxylic acid (1.74 mmol), Se powder (3.48 mmol), NaBH_4_ (7.1 mmol), and ethyl iodide (3.48 mmol). Conditions: 45 min reaction with NaHSe and 2 h reaction with ethyl iodide. A yellow solid was obtained, which was purified through column chromatography using ethyl acetate/hexane (1:10) as the eluent. Yield: 10%; m.p.: 35–36 °C. ^1^H NMR (400 MHz, CDCl_3_): δ 1.5 (t, 6H, 2–CH_3_, *J*_CH2-CH3_ = 7.5 Hz), 3.11 (q, 4H, –CH_2_–), 7.17 ppm (s, 2H, H_3_ + H_4_). ^13^C NMR (100 MHz, CDCl_3_): δ 15.8 (–CH_3_), 19.2 (–CH_2_–), 114.7 (C_3_ + C_4_), 153.5 (C_2_ + C_5_), 183.5 ppm (–C=O). IR (KBr): ν 3143 (w, C−H_arom_), 2961–2860 (s, C−H_aliph_), 1649 cm^−1^ (s, −C=O). HRMS calculated for C_10_H_13_O_3_Se_2_ (M + H): 340.91896; found: 340.91891.

##### Pentyl 3-Chlorothiophen-2-Carboselenoate (**1b**)

From 3-chlorothiophen-2-carboxylic acid (2 mmol), Se powder (2 mmol), NABH_4_ (2.15 mmol), and pentyl iodide (2.15 mmol). Under N_2_ atmosphere, absolute ethanol (10 mL) was added to a mixture of NaBH_4_ and selenium cooled by an ice bath, with magnetic stirring. After the formation of NaHSe was achieved, the ice bath was removed and the following reactions were carried out at room temperature. Before adding an excess of pentyl iodide, the mixture was filtered. Conditions: 20 min reaction with NaHSe and 20 min reaction with pentyl iodide. The solvent was eliminated under rotatory evaporation. The product was purified through column chromatography using a gradient elution of ethyl acetate: hexane. An orange oil was obtained. Yield: 53%. ^1^H NMR (400 MHz, CDCl_3_): δ 0.83 (t, 3H, –CH_3_, *J*_CH3-CH2_ = 7.1 Hz), 1.27–1.35 (m, 4H, γ + δCH_2_), 1.66–1.77 (m, 2H, ßCH_2_), 3.02 (t, 2H, αCH_2_, *J*_CH2-CH2_ = 7.4 Hz,), 6.96 (d, 1H, H_4_, *J*_4-5_ = *J*_5-4_ = 5.3 Hz), 7.44 ppm (d, 1H, H_5_). ^13^C NMR (100 MHz, CDCl_3_): δ 12.94 (−CH_3_), 21.19 (δCH_2_), 25.56 (αCH_2_), 28.89 (ßCH_2_), 31.18 (γCH_2_), 126.82 (C_4_), 129.50 (C_2_), 129.76 (C_5_), 136.56 (C_3_), 183.18 ppm (−CO). IR (KBr): ν 2922 −2852 (s, C−H_alif_), 1669 cm^−1^ (s, −C=O).

##### Dibenzyl 2,5-Furandicarboselenoate (**2c**)

From 2,5-furandicarboxylic acid (1.74 mmol), Se powder (3.48 mmol), NABH_4_ (7.1 mmol), and benzyl bromide (3.48 mmol). Conditions: 1.5 h reaction with NaHSe and 3.5 h reaction with benzyl bromide. The product was extracted with methylene chloride, further washed with water, dried over Na_2_SO_4._ The solvent was eliminated under rotatory evaporation. A yellow oil was obtained, which was precipitated and washed with diethyl ether. A yellow solid was obtained. Yield: 25%; m.p.: 114–115 °C. ^1^H NMR (400 MHz, CDCl_3_): δ 4.34 (s, 4H, 2–CH_2_–), 7.2 (s, 2H, H_3_ + H_4_), 7.22–7.35 ppm (m, 10 H, H_arom_). ^13^C NMR (100 MHz, CDCl_3_): δ 28.5 (–CH_2_–), 115.0 (C_3_ + C_4_), 127.3 (C_4’_), 128.7 (C_2’_ + C_6’_), 129.1(C_3′_ + C_5′_), 138.21 (C_1’_), 153.2 (C_2_ + C_5_), 182.8 (−C=O). IR (KBr): ν 3123−3088 (s, C−H_arom_), 1677 cm^−1^ (s, −C=O). HRMS C_20_H_16_O_3_Se_2_Na (M + Na^+^): calculated 486.9322; found 486.9430. 

#### 4.9.2. Procedure for Compound **3**

Compound **3** was synthesized in a previous work [32], under the reference **3c.**

### 4.10. Timelapse

0.5 × 10^6^ cells were seeded in 25 cm^2^ flasks and incubated at 37 °C under 5% CO_2_ 24 h. Media was changed and cells were treated with 5 µM of compounds for 24 h, after which floating cells were collected, centrifuged, and seeded at a density of 50,000 cells in total volume of 100 µL/well in a 96-well plate. Post 48 h of treatment, the cells were imaged live for another 24 h in Operetta and images were captured every 5 min. 

### 4.11. Confocal

Monolayer cultures were stained with CellTracker Red or Green (Invitrogen) for 1 h at 37 °C in the absence of serum. After this, 0.4 × 10^5^ cells stained with each of the cell trackers were mixed (1:1) and seeded onto 25 cm^2^ flasks for 24 h, followed by treatment with 5 µM of respective compounds for 18 h. Cells were imaged live using an LSM 800 confocal microscope (Zeiss, Oberkochen, Germany).

### 4.12. Statistical Analysis

One-way ANOVA followed by Dunnet’s test was performed using GraphPad 6.01 (GraphPad Software, San Diego, CA, USA). (* *p* < 0.05, ** *p* < 0.01, *** *p* < 0.001). 

## Supplementary Materials

Supplementary materials can be found at http://www.mdpi.com/1422-0067/19/10/2849/s1.

## Abbreviations

BznSeH	Benzeneselenol
CatB	Cathepsin B
CatD	Cathepsin D
CD29	β1-integrin
CDC42	Cell division control protein 42 homolog
PARP	DNA damage-responsive enzymes poly(ADP-ribose) polymerase
MSA	Methylseleninic acid
MeSeCys	Methylselenocysteine
CH_3_SeH	Methylselenol
Nrf2	nuclear factor erythroid 2 related factor 2
PDAC	Pancreatic ductal adenocarcinoma
PI	Propidium iodide
ROS	Reactive oxygen species
Se	Selenium
