# Supplementary material for: Novel Methylselenoesters Induce Programed Cell Death via Entosis in Pancreatic Cancer Cells
Source: Int J Mol Sci. 2018 Sep 20;19(10):2849. doi: 10.3390/ijms19102849 (PMC6213452; doi:10.3390/ijms19102849)
Supplement: Supplementary file 1 [file ijms-19-02849-s001.zip › Khalkar et. al._Supplementary Files/ijms-355725-Supplementary Chemical Information.pdf]

SUPPLEMENTARY CHEMICAL INFORMATION

^{1}H NMR of compound **1a**

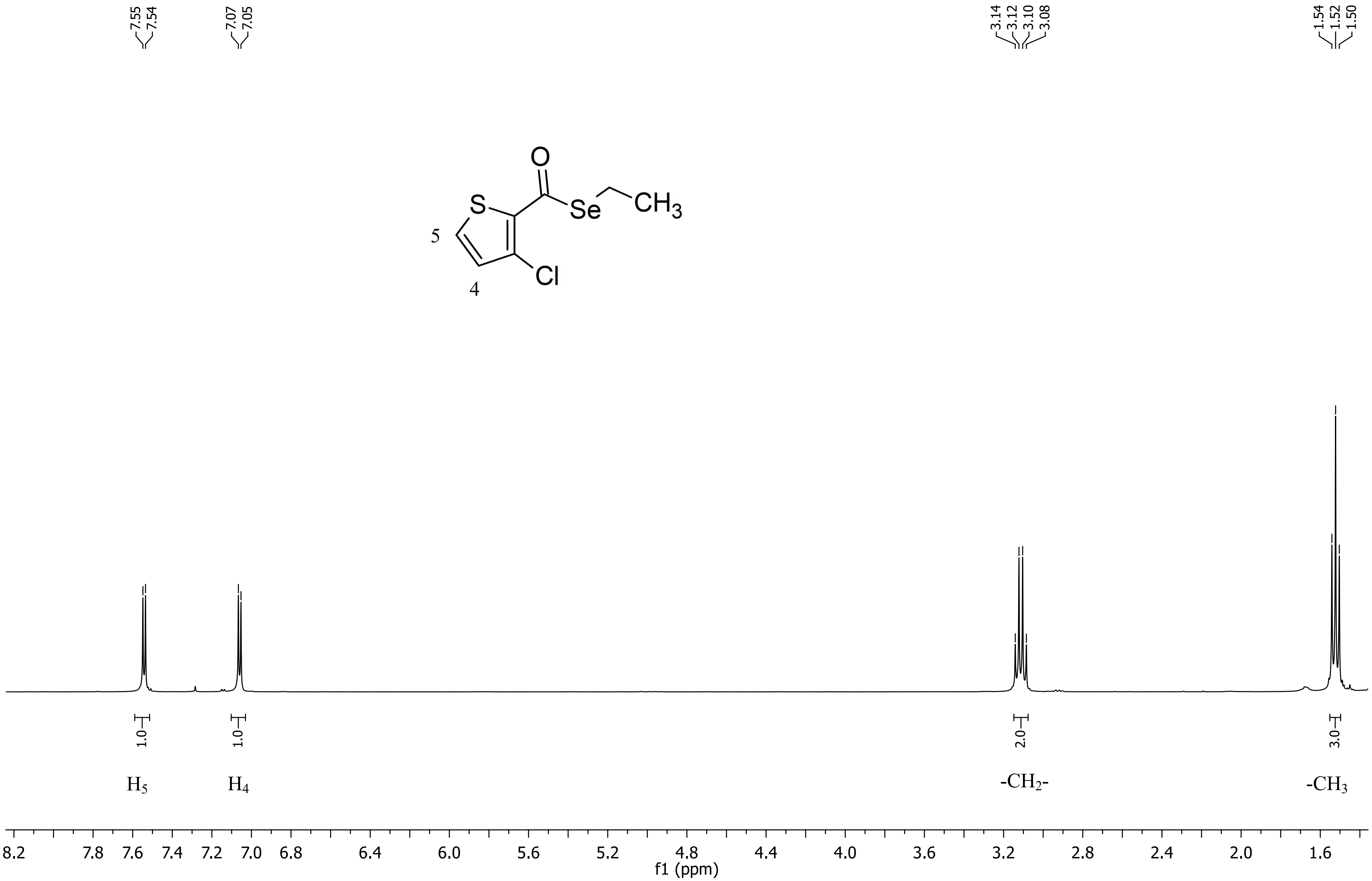

^{13}C NMR of compound **1a**

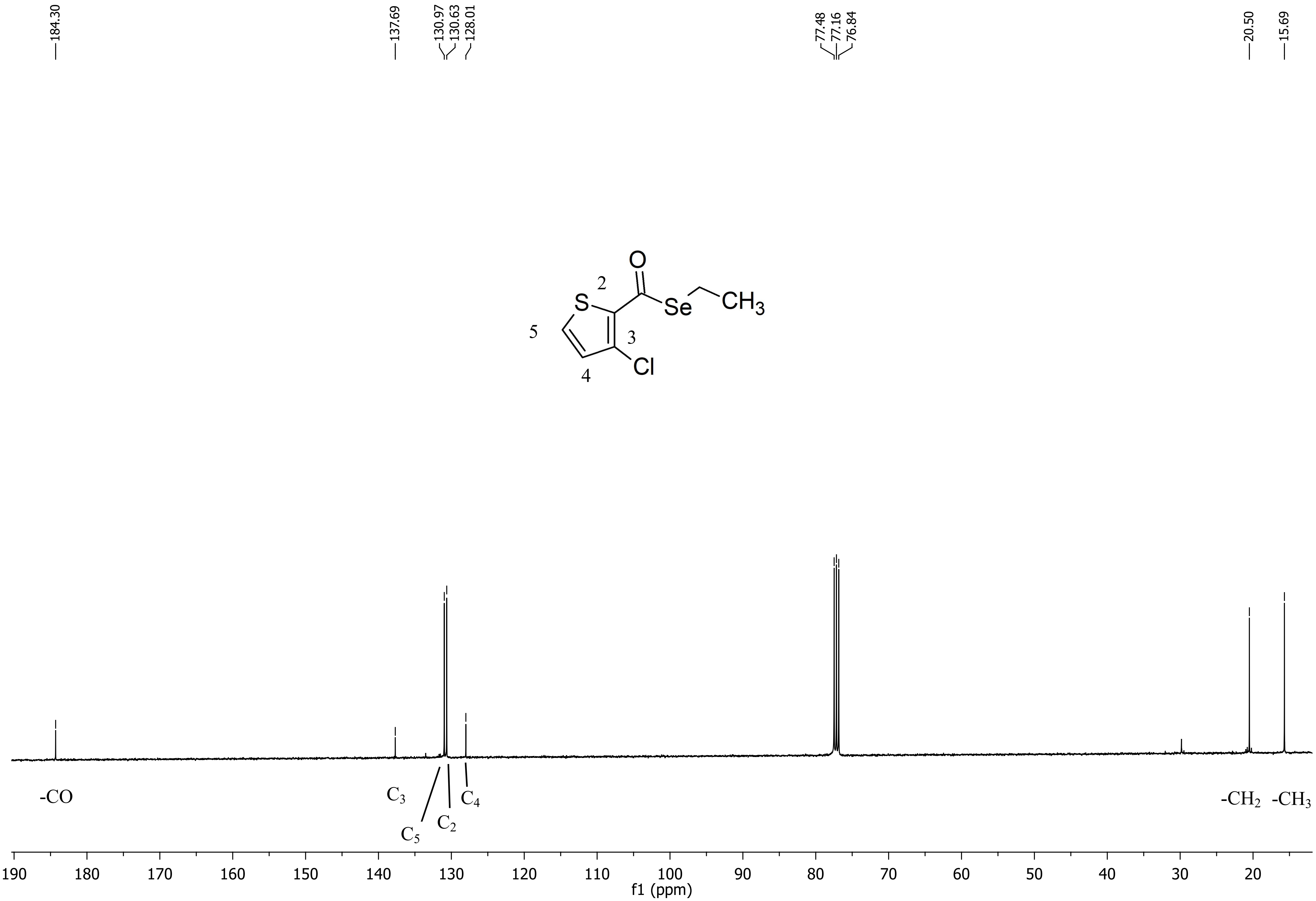

^{1}H NMR of compound **1b**

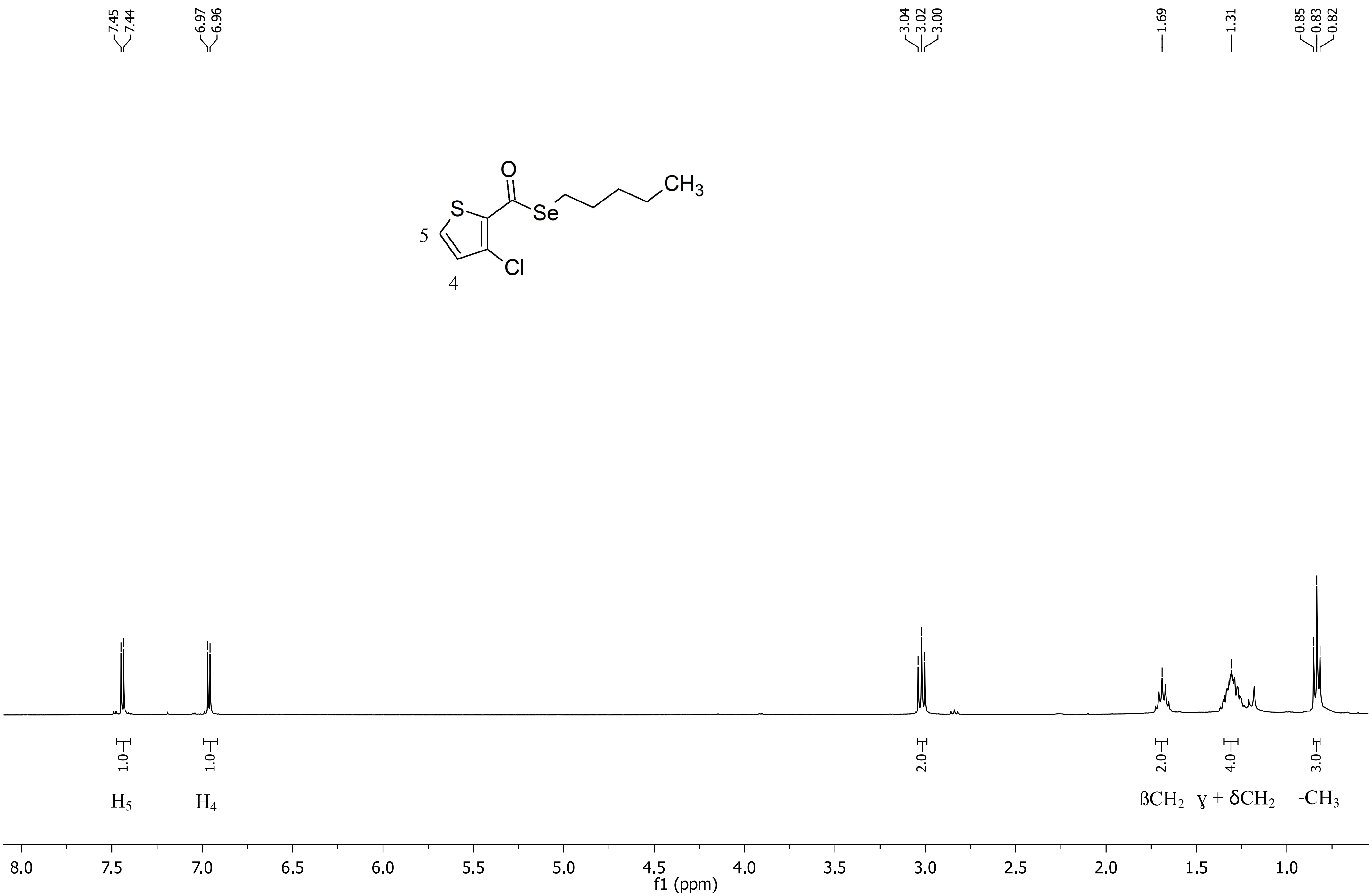

^{13}C NMR APT of compound **1b**

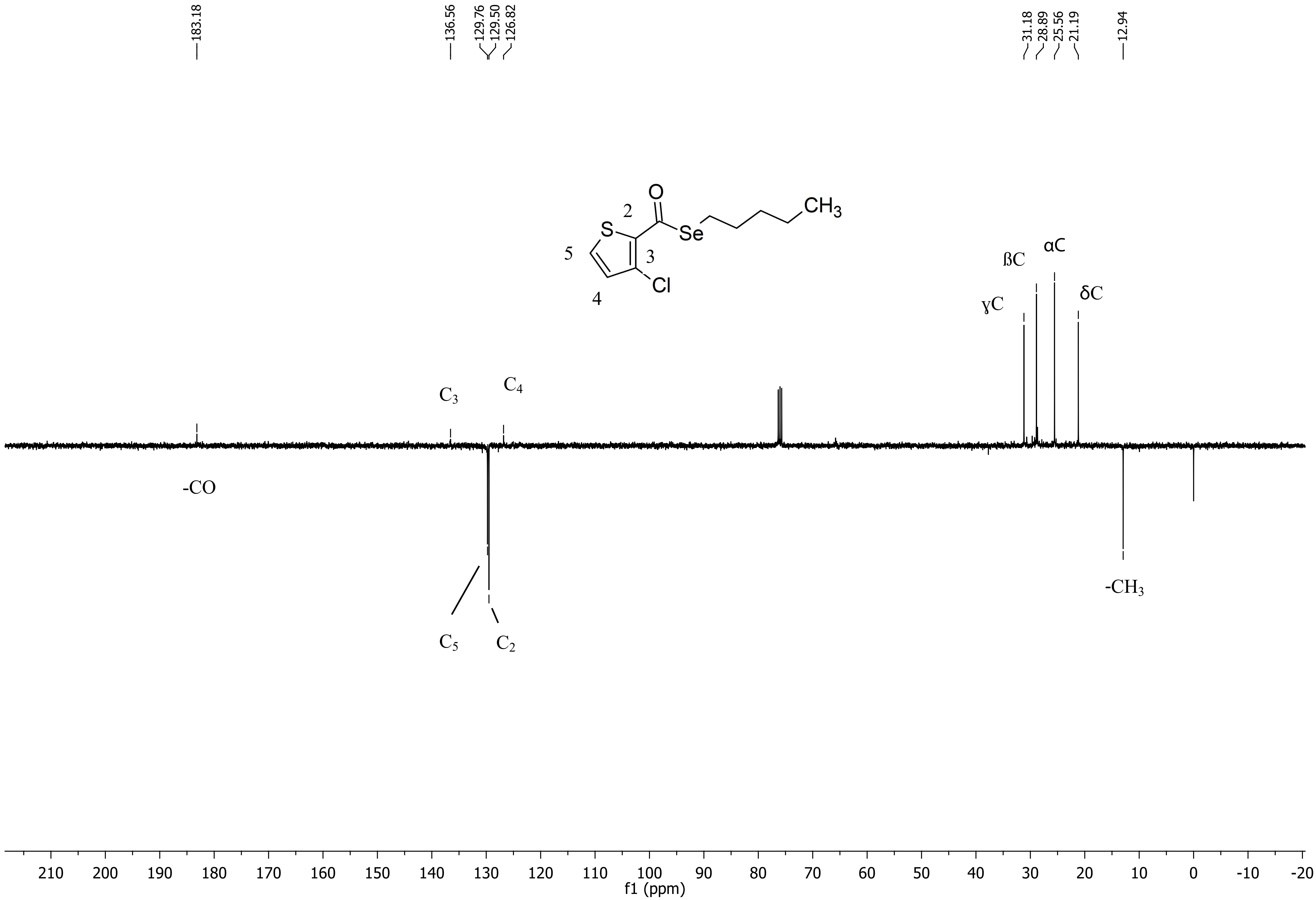

^{1}H NMR of compound **2a**

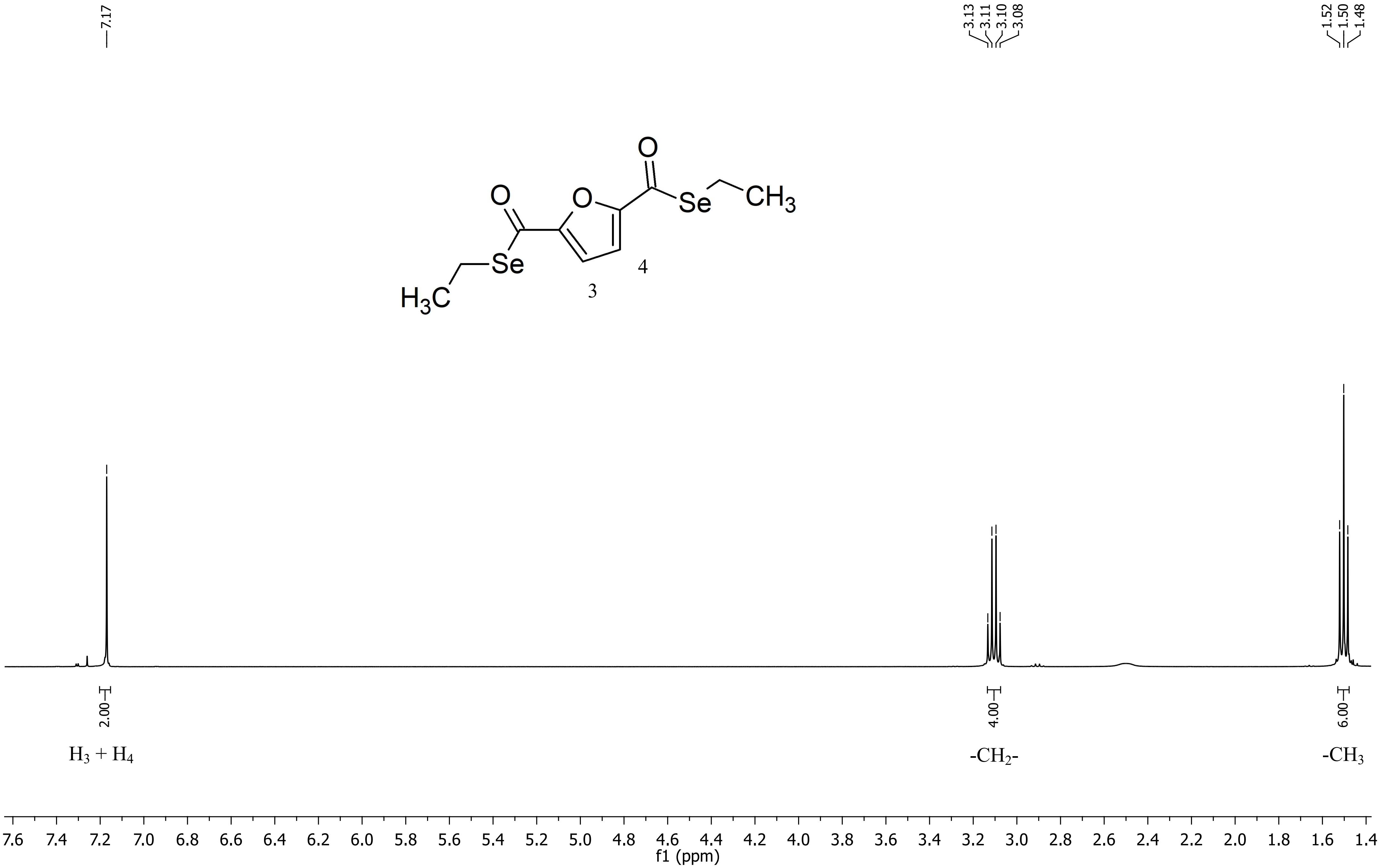

^{13}C NMR of Compound **2a**

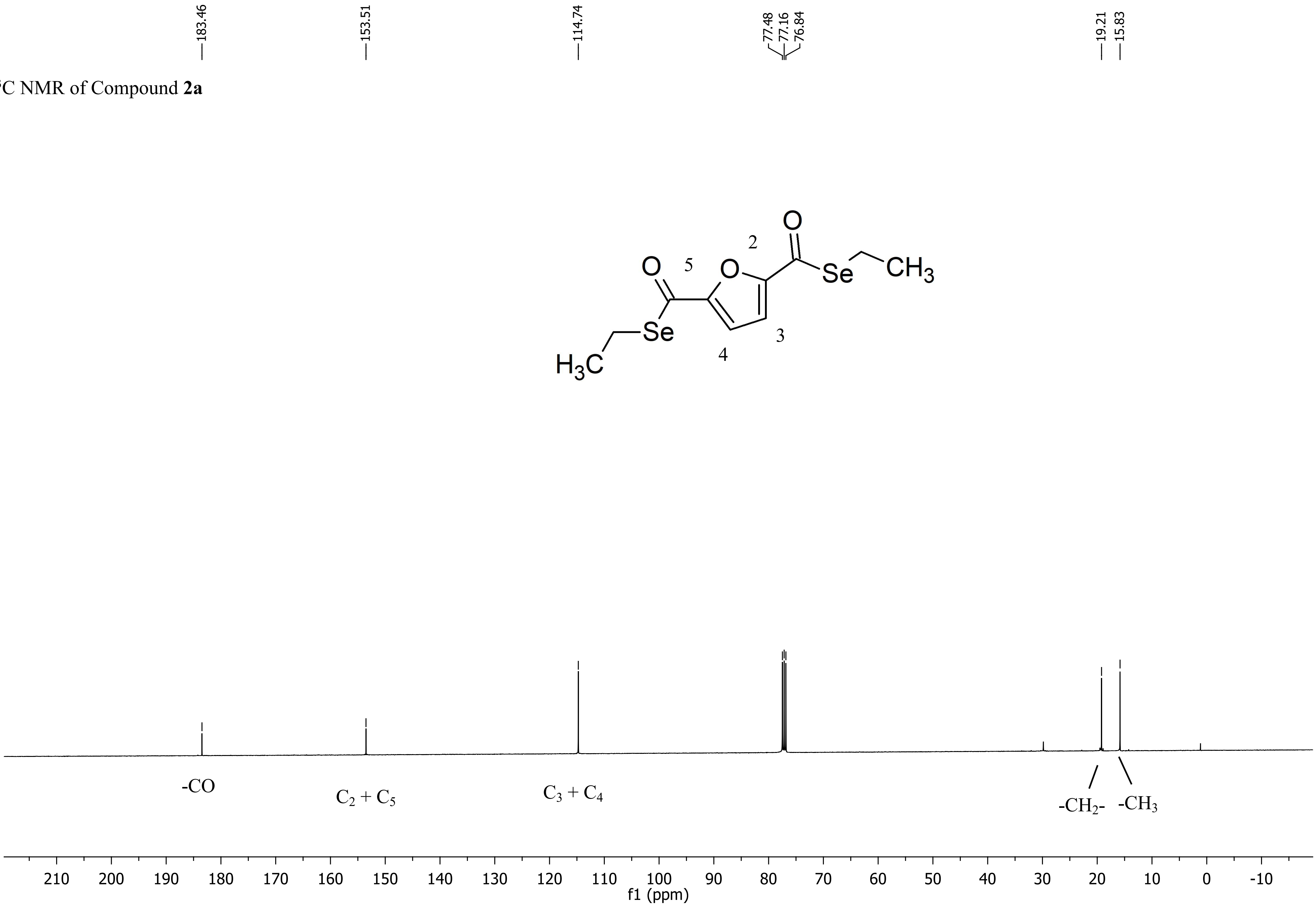

^{1}H NMR of compound **2c**

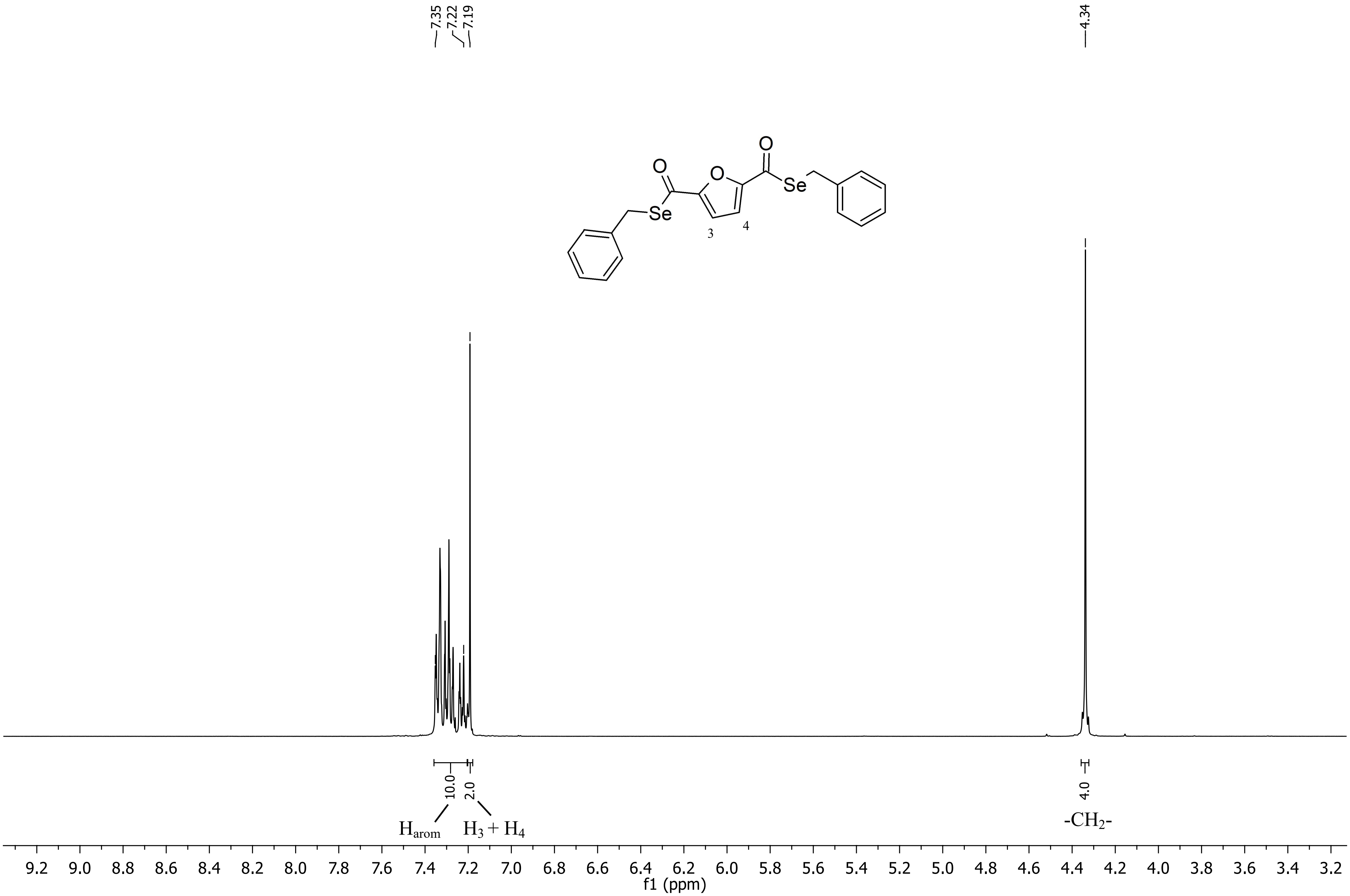

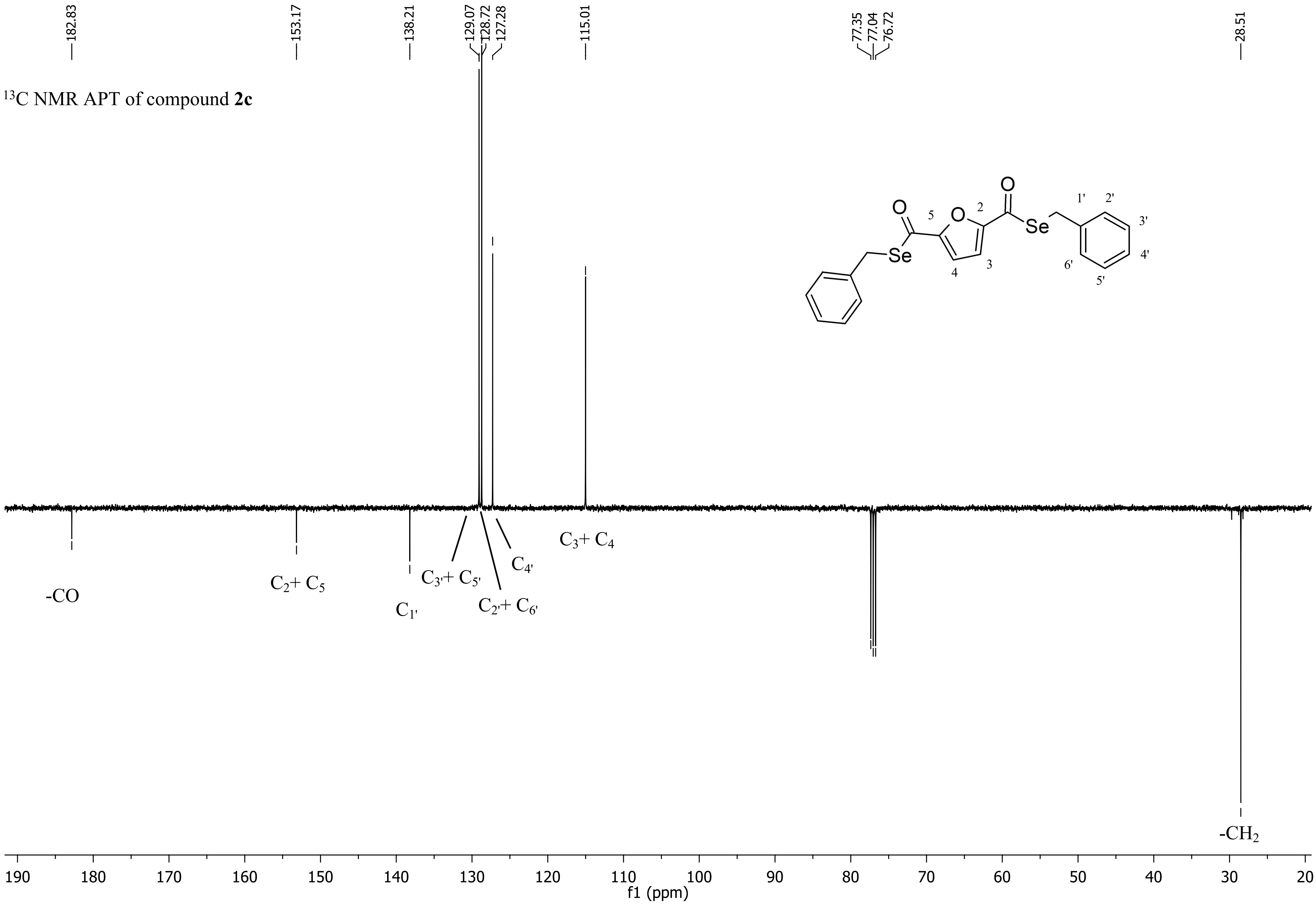

182.83
153.17
138.21
129.07
128.72
127.28
115.01
77.35
77.04
76.72
28.51
^{13}C NMR APT of compound **2c**
O
O
5
O
2
Se
Se
1'
2'
3'
4'
5'
6'
4
3
-CO
C_2+ C_5
$C_{1'}$
$C_{3'}$+ $C_{5'}$
$C_{2'}$+ $C_{6'}$
$C_{4'}$
C_3+ C_4
-CH_2
190
180
170
160
150
140
130
120
110
100
90
80
70
60
50
40
30
20
f1 (ppm)
