# Supplementary material for: Novel Methylselenoesters Induce Programed Cell Death via Entosis in Pancreatic Cancer Cells
Source: Int J Mol Sci. 2018 Sep 20;19(10):2849. doi: 10.3390/ijms19102849 (PMC6213452; doi:10.3390/ijms19102849)
Supplement: Supplementary file 1 [file ijms-19-02849-s001.zip › Khalkar et. al._Supplementary Files/ijms-355725-Supplementary Figures.pdf]

Supplementary figure 1

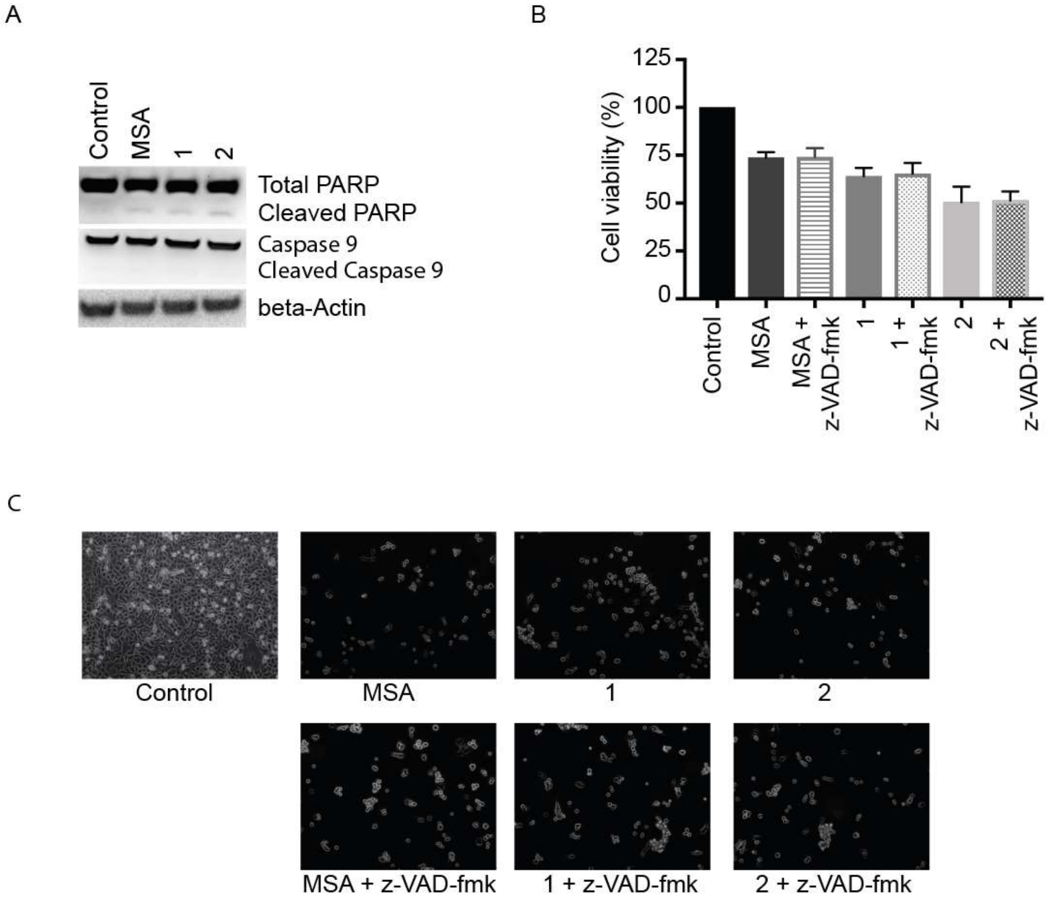


**Supplementary Figure 1**. MSA, compounds **1** and **2** induce a caspase-independent slight increase in cleaved PARP. (A) Western blot analysis of PARP and caspase 9 levels after 72 h and 48 h treatment with the compounds respectively. (B) Cell viability assessed by Trypan blue exclusion after treatment with the compounds in the presence of the pancaspase inhibitor z-vad-FMK at 72 h.

**Supplementary videos.** Post 24 h of seeding, Panc-1 cells were treated with **MSA** (**video 1**) or compound **1** (**video 2**) or compound **2** (**video 3**) for 48 h, after which the floating cells were plated at a density of 50000 cells per well of a 96 well plate pre-coated with polyhema. Timelapse microscopy showing the process of entosis, where upon treatment with respective compounds, one cell engulfs another cell further leading to its degradation.
