# Supplementary figures and images for: Novel Methylselenoesters Induce Programed Cell Death via Entosis in Pancreatic Cancer Cells
Source: Int J Mol Sci. 2018 Sep 20;19(10):2849. doi: 10.3390/ijms19102849 (PMC6213452; doi:10.3390/ijms19102849)

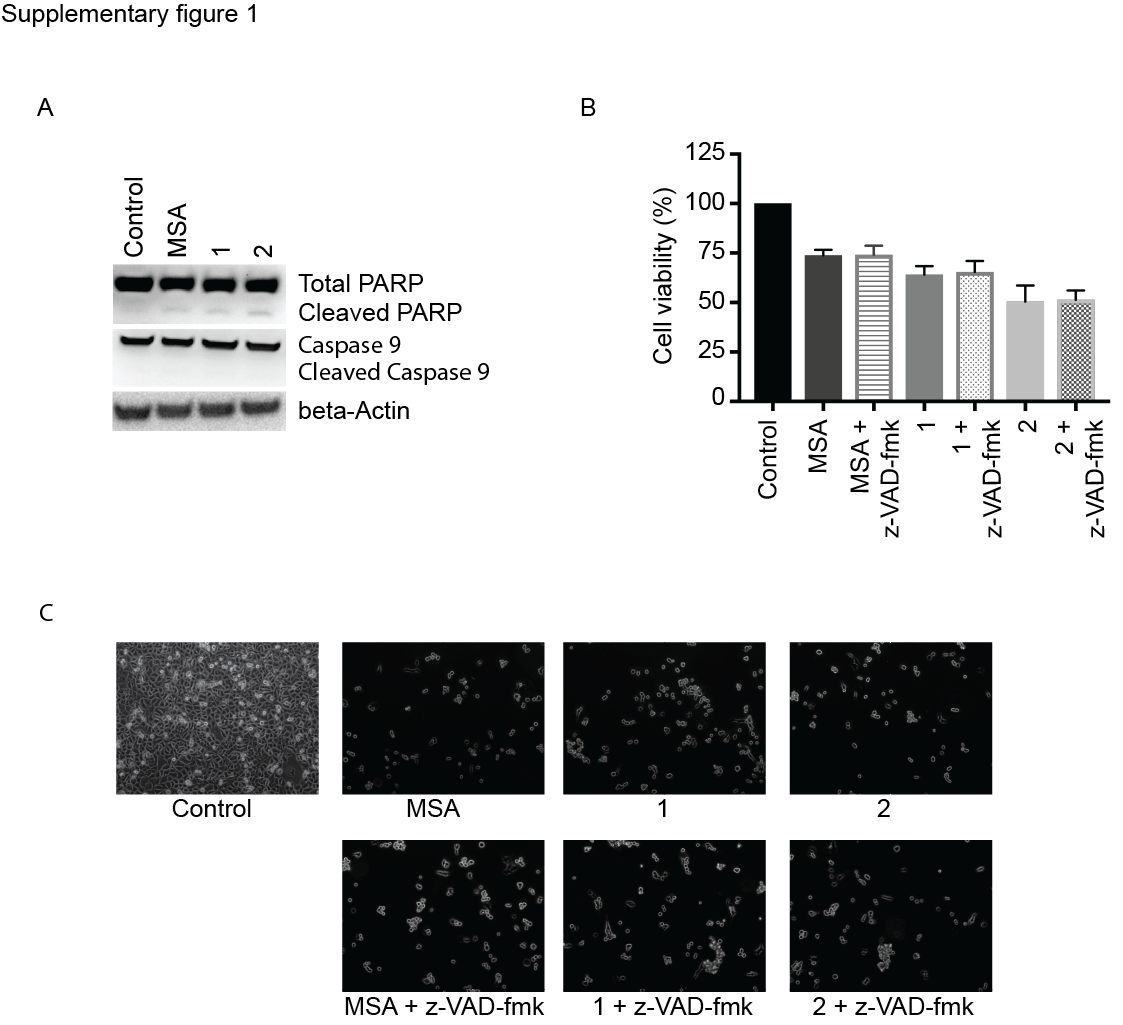

Supplement: Supplementary file 1 [file ijms-19-02849-s001.zip › Khalkar et. al._Supplementary Files/Khalkar et. al._Supplementary Figure 1.tif]
